# Supplementary material for: Sociotechnical imaginaries of autonomous vehicles: Comparing laboratory and online eye-tracking methods
Source: PLoS One. 2025 Nov 17;20(11):e0335672. doi: 10.1371/journal.pone.0335672 (PMC12622791; doi:10.1371/journal.pone.0335672)
Supplement: S1 Appendix — (DOCX) [file pone.0335672.s001.docx]

**APPENDIX 1**

**Table 8.** Planned online sample and final online sample

| **Categories** | **Sub-categories** | **No. of Cities Wanted** | **No. of Cities Completed** | **No. of Participants Wanted** | **No. of Participants Completed** |
| --- | --- | --- | --- | --- | --- |
| Experience with AV pilots | High and medium CIMI Ranked | 10 | 15 | 1000 | 534 |
| No experience with AV pilots | Low CIMI Ranked | 10 | 11 | 1000 | 737 |
| **Total** | | **20** | **26** | **2000** | **1276*** |

*Note: Data, about the city they come from, are not available for 5 participants*
