# Supplementary material for: Sociotechnical imaginaries of autonomous vehicles: Comparing laboratory and online eye-tracking methods
Source: PLoS One. 2025 Nov 17;20(11):e0335672. doi: 10.1371/journal.pone.0335672 (PMC12622791; doi:10.1371/journal.pone.0335672)
Supplement: Attachment — Submitted filename: Rebuttal Letter.pdf [file pone.0335672.s003.pdf]

Dear editors,

We would like to express our sincere gratitude for the valuable comments on our article ‘Investigating Sociotechnical Imaginaries of Autonomous Vehicles: Comparing Laboratory and Online Eye-Tracking Methodology’.

The reviewers’ comments have helped us to significantly improve our article. Please find attached our revised article and the comments on the reviewers’ suggestions. We have marked the changes in the article using ‘Track changes”, and our responses to reviewers’ comments are below in blue color.

Thank you for considering our article for publication! We are looking forward to hearing soon about you!

Yours sincerely,

Mergime Ibrahimi
Anu Masso
Mauro Bellone

Review #1

This paper focuses on an interesting topic. Based on eye-tracking techniques, the authors investigate sociotechnical imaginaries of autonomous vehicles using both in-lab and online eye-tracking experiments. However, there are several issues to be clarified.

1) Page 4, Line 4. The authors need to review more up-to-date articles in Introduction section. Here are three studies for your information.

[1] A day in the life with an automated vehicle: Empirical analysis of data from an interactive stated activity-travel survey.

[2] Understanding Human Drivers' Trust in Highly Automated Vehicles via Structural Equation Modeling.

[3] Exploring the association between socio-demographic factors and public acceptance towards fully automated vehicles: Insights from a survey in Australia.

We are highly thankful for this valuable suggestion. We have reviewed the studies mentioned and incorporated them into the Introduction section. Specifically, we have added references [1], [2], and [3] to the introduction on page 4, lines 64-72, where we highlight recent advancements in automated vehicle research. This addition has strengthened the context and relevance of the study within the current literature.

2) Page 8, Lines 145 to 146. The relationship between driver trust on autonomous vehicles and their attention during autonomous driving is also an important factor for traffic safety and public acceptance. See the reference "Latent Hazard Notification for Highly Automated Driving: Expected Safety Benefits and Driver Behavioral Adaptation."

We appreciate the reviewer's insightful comment. While trust in autonomous vehicles has been studied extensively, our paper focuses on the perception of individuals towards autonomous vehicles, particularly how imaginaries are formed based on the experience and information available to them. Perception, in this context, is shaped by external narratives and information, which not only influences, but also plays a role in, the creation of trust. Trust, however, tends to develop more gradually through direct experience, whereas perception is shaped by the information individuals receive and how they interpret it.

That being said, we acknowledge the relevance of the suggested study to our broader discussion on safety and public acceptance which shapes people's imaginaries and perception. As such, we have included the suggested reference on page 8, lines 183-188, where we elaborate on the relationship between trust and driver behavior during automated driving. This addition provides further depth to the discussion on public acceptance.

3) Page 11, Lines 226 to 227. Why not invite automotive engineers/ scientists for the in-lab experiment? As these groups of people are the developer and tester of autonomous vehicles.

We appreciate the reviewer's suggestion. In fact, our in-lab participants included engineers and scientists from the field of Computer Science (besides Social Scientists), many of whom have hands-on experience with autonomous vehicles and algorithms. Additionally, several participants have been actively involved in studying and evaluating algorithms, providing further depth to their insights. Their technical expertise in relevant areas allowed us to capture valuable perspectives on both the development and testing processes of autonomous vehicles, which enriched the outcomes of our study. Furthermore, besides engineers and scientists from Computer Science, including Social Scientists in our sample enabled us to capture insights that extended beyond technical considerations to encompass potential societal impacts. We clarified this on page 12, lines 294-302.

4) Page 12, Line 233. The authors should give a description on the list of cities and the corresponding languages.

We appreciate the reviewer's comment. In response, we added in the endnote (page 44) the cities from which participants were drawn. The experiment itself was conducted in English. Given that our participants are rather digitally aware and literate, along with the global spread of the CINT platform – where panel members are frequently engaged in international studies conducted in English – we believe that language did not pose any significant barriers during the experiment.

5) Page 12, Table 1. The authors should report the respondents' experience of using autonomous driving systems.

We appreciate the reviewer's suggestion, and we agree that this is an important aspect to report. In response, we have added the collected data on participants' trust in and willingness to try fully automated autonomous vehicles to Table 1 (see page 14). This additional information provides further insight into the respondents' perception toward autonomous driving systems, which is essential for understanding their perspectives in our study.

6) Page 12, Table 1. How do the authors categorize the educational levels?

We appreciate the reviewer's comment. In response, we have added a legend in Table 1 (page 14) that explains the categorization of educational levels into Basic, Secondary, and Higher categories. However, for clarity and consistency, we followed international studies such as the European Social Survey. Considering the varying education categories used in RealEye platform and CINT for different cities, we merged some of the categories to ensure efficiency. Therefore, we propose the following simplified categories:

- **Basic** includes early childhood education, primary education, and lower secondary education.

- **Secondary** includes upper secondary education and post-secondary non-tertiary education.
- **Higher** includes short-cycle tertiary education, bachelor's degrees, master's degrees, and doctoral degrees.

7) Page 18, Lines 348 to 351. It would be clearer and more understandable if the authors summarize the hypotheses of their study in the previous sections (e.g., Section 3). Moreover, all research hypotheses should be numbered.

We appreciate the reviewer's thoughtful suggestion. Our study is exploratory in nature, however, we recognize the value of this feedback and have now formulated more detailed research questions and assumptions regarding expected results. These have been included in Section 1 on page 5 to provide clearer guidance on the objectives and focus of the study.

8) Pages 19 and 20, Table 3 and Table 4. The authors should report p-values, rather than only reporting t-values.

For Table 3, we have added a note in the legend indicating that each scenario category showed $p < 0.001$ between AV and non-AV settings, and a detailed explanation on page 22 in the text lines 479-524. Regarding Table 4, we would like to clarify that the sample size was relatively small, which limited our ability to draw definitive conclusions. Therefore, we have added the explanation in the Table 4 legend and further explanation on page 22 in the text lines 525-532.

Review #2

This paper examines the sociotechnical imagination of autonomous vehicles and compares the differences in cognitive engagement between laboratory and online eye-tracking methods. The research objectives are clear, namely to compare the differences between laboratory and online eye-tracking methods in studying the sociotechnical imagination of autonomous vehicles. The research methods are scientific, the data analysis is thorough, and the results are innovative and practically meaningful. However, there is still room for improvement in aspects such as sample selection and discussion of results.

Simplify the Background Summary: The current background section is relatively lengthy. It can be streamlined to summarize existing research more concisely, highlighting the innovative points of this study. It is recommended to add a concise description of the research questions and hypotheses in the introduction. The background section provides a detailed overview of research on autonomous vehicle technology and its societal impacts. It is suggested to summarize the main findings of existing research more briefly in the introduction and clearly point out the innovations of this study.

We appreciate the reviewer’s suggestion to streamline the background section. In response, we have revised this section to provide a more concise summary of existing research. We have also emphasized the innovative aspects of our study to make these contributions clearer. Additionally, we have included a brief overview of our research questions and assumptions for expected results in the introduction, guiding the reader through the study’s purpose (Section 1, page 5).

The article describes the experimental design in detail, including specific operational steps for laboratory and online experiments, sample structure, scenario design, etc. Sufficient explanations are also provided for experimental equipment and technical details. However, it needs to more clearly explain the rationality and representativeness of sample selection, especially how to ensure sample diversity and representativeness in online experiments.

We appreciate the reviewer’s comment on the importance of explaining sample selection and representativeness more clearly. In our online experiments, we ensured diversity by selecting participants from cities with varying socio-demographic backgrounds, as well as cities that have hosted AV pilots, based on the AV Initiatives Cities © 2017 Bloomberg Philanthropies list. Additionally, we referred to the CMI Rank 2020 to ensure inclusion of cities with both high and low rankings in terms of technological readiness and development. We have added a table in the manuscript Appendix 1 to present the initial online sample plan and the final sample (page 44).

Additionally, we utilized the CINT platform, which allows us to choose the sample based on the proportion of the national census. This ensures that the online sample is representative of the population in terms of key demographic variables (e.g., age, gender, education level, etc.). By

leveraging the platform's capabilities, we were able to enhance the representativeness and diversity of the sample, providing a reliable basis for our analysis. We have added a further explanation on page 13, lines 308-325, and page 14, lines 340-345. Although our goal was to recruit participants based on their respective city census data, it is important to note that online participants tend to be younger and often possess higher educational backgrounds. This sample skewness could result in an over-representation of views from more educated individuals and an under-representation of older people. We have added such limitations in Section 5.1, page 34, line 804-814.

By comparing gaze duration, reaction time, and other indicators between laboratory and online experiments, the article analyzes the differences in cognitive engagement between the two methods. It is recommended to add more statistical tests in the data analysis section to verify significant differences in gaze behavior among different groups (such as algorithm-aware and non-aware individuals). Additionally, detailed analysis of different scenario categories can be included.

We thank the reviewer for this valuable suggestion. In response, we have added additional statistical tests (Table 5, page 24) to the data analysis section to verify significant differences in gaze behavior between the groups and scenario settings, specifically comparing data&algorithm-informed and uninformed individuals. These additional tests are further elaborated on pages 24, lines 569-576, where we report the significance levels and discuss the findings.

Clearly describe the specific criteria and steps for sample selection in online experiments to ensure sample diversity and representativeness. Explain how the CINT platform is used to ensure sample representativeness and provide relevant statistical data.

We thank the reviewer for highlighting the need for more clarity on sample selection criteria and steps. In response, we have added a detailed explanation of specific criteria and steps for sample selection in the online experiment on page 13, lines 308-325, and outlined how we ensured sample diversity and representativeness in the online experiments on page 14, lines 340-345.

The article discusses the significance of the experimental results, pointing out the differences in data quality and participant behavior between laboratory and online experiments. The conclusion section summarizes the main findings of the study and suggests directions for future research. It is recommended to provide a deeper analysis of the potential reasons for the results in the discussion section, such as the psychological state of participants and the impact of environmental factors under different experimental conditions. Furthermore, the practical application value of the research and its implications for policy formulation can be further discussed.

We thank the reviewer for this valuable suggestion. We sought to control the environment by selecting participants who were able to run the experiment via laptops rather than mobile phones. This decision aimed to reduce movement during the experiment, as individuals are generally more stationary when using laptops, which improves the accuracy of eye-tracking data. At the individual level, however, it was more challenging to monitor environmental conditions and psychological states, as the experiments were conducted online without direct supervision from researchers. We acknowledge this as a limitation, as factors such as distractions, lighting, and participant focus could not be fully controlled. This aspect is discussed as a potential drawback of the online method in comparison to in-lab experiments, page 35, lines 815-819.

Additionally, we have expanded our discussion on the practical applications of the research and its implications for policy formulation, providing more detail on how our findings can inform future developments in AV technologies, page 34, lines 790-797.

Some paragraphs in the article are relatively long and can be appropriately segmented to make the content more readable. For certain technical terms, annotations or explanations can be added to help readers better understand.

We have revised the manuscript to improve readability by segmenting longer paragraphs, making the content easier to follow. Additionally, we have added further explanations for technical terms, specifically, we have added legends in the tables to ensure that all readers can fully understand the concepts discussed. These changes are reflected throughout the text, and we believe they enhance the clarity and accessibility of the paper.
