# Supplementary material for: Sociotechnical imaginaries of autonomous vehicles: Comparing laboratory and online eye-tracking methods
Source: PLoS One. 2025 Nov 17;20(11):e0335672. doi: 10.1371/journal.pone.0335672 (PMC12622791; doi:10.1371/journal.pone.0335672)
Supplement: Attachment — Submitted filename: Response to Reviewers.docx [file pone.0335672.s004.docx]

Dear Dr. Quan Yuan,

We would like to express our sincere gratitude for the valuable comments on our article *‘Sociotechnical Imaginaries of Autonomous Vehicles: Comparing Laboratory and Online Eye-Tracking Methods’.*

The reviewers’ constructive feedback has significantly improved our article. We have carefully addressed all comments and suggestions, made the necessary revisions, and provided detailed explanations in the response letter. In addition, considering the journal’s high standards and the reviewers’ valuable input, we have made minor linguistic and phrasing refinements to enhance clarity. For instance, we removed a word from the title to improve comprehension. However, all substantive changes are solely those recommended by the reviewers.

We have also added a note indicating that the first two authors contributed equally, designating the second author as a co-first author. This formal addition acknowledges the second author’s role in initiating and conducting the study. The authors’ order remains unchanged.

Please find attached our revised article and responses to the reviewers’ suggestions. We have marked the changes using ‘Track Changes,’ and our responses are highlighted in blue.

Thank you for considering our article for publication. We look forward to your response.

Yours sincerely,

Mergime Ibrahimi

Anu Masso

Mauro Bellone

1. If the authors have adequately addressed your comments raised in a previous round of review and you feel that this manuscript is now acceptable for publication, you may indicate that here to bypass the “Comments to the Author” section, enter your conflict of interest statement in the “Confidential to Editor” section, and submit your "Accept" recommendation.

Reviewer #1: All comments have been addressed

Reviewer #2: All comments have been addressed

2. Is the manuscript technically sound, and do the data support the conclusions?

The manuscript must describe a technically sound piece of scientific research with data that supports the conclusions. Experiments must have been conducted rigorously, with appropriate controls, replication, and sample sizes. The conclusions must be drawn appropriately based on the data presented.

Reviewer #1: Yes

Reviewer #2: Yes

3. Has the statistical analysis been performed appropriately and rigorously?

Reviewer #1: Yes

Reviewer #2: Yes

4. Have the authors made all data underlying the findings in their manuscript fully available?

The [PLOS Data policy](http://track.editorialmanager.com/CL0/http:%2F%2Fwww.plosone.org%2Fstatic%2Fpolicies.action%23sharing/1/010f0193d3001546-b1869f74-6e02-4375-ad6a-3d70451136dd-000000/NWcRAzCkR1IslSCuC3mYUvteBbeSMcWk08BqrhNzASs=189) requires authors to make all data underlying the findings described in their manuscript fully available without restriction, with rare exception (please refer to the Data Availability Statement in the manuscript PDF file). The data should be provided as part of the manuscript or its supporting information, or deposited to a public repository. For example, in addition to summary statistics, the data points behind means, medians and variance measures should be available. If there are restrictions on publicly sharing data—e.g. participant privacy or use of data from a third party—those must be specified.

Reviewer #1: Yes

Reviewer #2: No

**Response**: We are deeply grateful for this feedback and fully understand the requirements of the PLOS Data Policy. We highly value and support the principles and ideals of open science. To meet these requirements, we have made every effort to make the data accessible to other researchers.

However, to ensure the confidentiality and anonymity of the respondents—as recommended by the research ethics committee—we have taken specific measures to balance the openness of science with the protection of research subjects. This is particularly important given the context of our study: a small-scale study with a limited number of participants who could potentially be identified, and a large-scale study addressing the evolving international regulations concerning platform workers’ rights.

To achieve this balance, we have taken the following concrete steps:

1. **Meta-data descriptions**: We have prepared thorough metadata descriptions to provide detailed explanations of the methodology used in the study. These metadata are openly available here: [<https://doi.org/10.5281/zenodo.13919434>](https://doi.org/10.5281/zenodo.13919434) .
2. **Data access request process**: Researchers who wish to reuse the data can request access from the authors. In such cases, a confidentiality agreement will be established between the researchers, in line with the data processing principles required by the research ethics committee.

We are committed to finding solutions that uphold the ideals of open science while protecting the rights and privacy of research participants. The steps highlighted above have also been updated in the PLOS ONE submission system.

5. Is the manuscript presented in an intelligible fashion and written in standard English?

PLOS ONE does not copyedit accepted manuscripts, so the language in submitted articles must be clear, correct, and unambiguous. Any typographical or grammatical errors should be corrected at revision, so please note any specific errors here.

Reviewer #1: Yes

Reviewer #2: Yes

**Response:** We are grateful for this positive feedback. To meet the highest standards, we have revised the article once again, focusing on grammar, style, and the overall smoothness of the text, making a few minor corrections. However, these corrections are purely linguistic, and the content has only been revised in the specific areas where the reviewers requested changes.

6. Review Comments to the Author

Please use the space provided to explain your answers to the questions above. You may also include additional comments for the author, including concerns about dual publication, research ethics, or publication ethics. (Please upload your review as an attachment if it exceeds 20,000 characters)

Reviewer #1: (No Response)

Reviewer #2: Through the revision of the article, the quality of the paper has been improved to some extent, but there are still some deficiencies in logic and result analysis. The following are some suggestions for improvement:

1. Is the questionnaire used in P12 a questionnaire that has been studied and demonstrated? If not, is the questionnaire tested for its validity, such as reliability and validity?

**Response**: We thank the reviewer for this question. We used some socio-demographic variables from the survey, such as participants’ country, education level, readiness to use AVs, and prior participation in similar studies, as part of our analysis framework. These variables were collected to contextualize viewing behavior, and are now explained in Section 3, pages 11-12, lines 332-343.

However, all the variables we collected with the survey were not analyzed or demonstrated in this paper, considering the methodological focus of this study. This article primarily focuses on analyzing viewing behavior, comparing two methods, online and in-lab eye-tracking, to contribute to the gaps in previous studies on social imaginaries about automated decision-making systems. Therefore, due to the space limitations of this article, the more in-depth analysis of associations between viewing behavior and background variables will remain the focus of future research. We are very thankful for this suggestion, and we have now explained this aspect in greater detail in the text. You can find the updated explanation in Section 3, page 11-12, lines 343-349.

In regard to validation, the questions were formulated based on validated instruments and frameworks from prior studies. For example, some questions regarding values toward algorithmic solutions were drawn from previously validated studies e.g. Masso, Kaun & van Noordt in 2024. Additionally, we conducted qualitative studies in earlier phases of our research (e.g., Masso & Kasapoglu, 2020) and relied on existing frameworks to formulate formalized questions in this survey. Moreover, during in-lab data collection, in-depth introductory and follow-up interviews were conducted, where participants were also asked to reflect on the experiment and the survey. It gave us an additional opportunity to ensure the reliability of the developed study instruments through cognitive testing of the survey instruments and ensure that the respondents’ understanding of the survey instruments corresponded with the researchers’ ideas. Additionally, test trials have been conducted before in-lab and online study. You can find the updated explanation in Section 3 on page 11, lines 320-333, and page 12, lines 354-358 so that it can be reviewed more easily.

2. Check whether the conclusions drawn from the analysis of DAI and DAU on page P25 are correct "For Dai participants, classified as such due to their prior participation in similar studies and presumed familiarity with algorithmic systems the average fixation duration (the depth of fixating in one point) and total fixation duration is higher in AV. For DAI, the t-test revealed that there was no statistically significant difference in the fixation durations between AV and non-AV scenarios, t(7080)=1.95, p=0.051. DAU participants showed the same results longer average fixation duration and total fixation duration longer towards AV. For DAU, the t-test indicated that there was a statistically significant difference in the fixation durations between AV and non-AV scenarios, t(10748)=2.11, p=0.034.”？

**Response**: We thank the reviewer for pointing out the need to confirm the accuracy of our conclusions based on the reported statistical results. To address this, we have revised the conclusions in the text to ensure alignment with the statistical findings. Specifically:

- For the DAI group, we clarified that while there was a trend towards longer fixation durations in AV scenarios, the result was not statistically significant (t(7080) = 1.95, p = 0.051).
- For the DAU group, we confirmed that the fixation durations were statistically significantly longer in AV scenarios (t(10748) = 2.11, p = 0.034), and this conclusion remains consistent with the data.

The revised text now accurately reflects these findings and avoids overgeneralization for the DAI group. This revision is reflected in Section, 4.2.1, page 25, lines 630-636 of the manuscript.

3. A Two-Way ANOVA is used in P25, but it is not stated whether the data meets the conditions of using variance test, such as whether the data meets normal distribution, etc.

**Response**: Thank you for the highly valuable comment raised. After your review, we have conducted Shapiro-Wilk for fixation time data. The results indicated deviations from normality (p < 0.05), which is expected given the data’s inherent skewness due to its composition from 26 cities. Furthermore, Levene’s test showed significant differences in variances for some fixation time categories, indicating that the assumption of homogeneity of variances is not fully met. Despite this, Two-Way ANOVA is robust to moderate deviations from normality and variance homogeneity, particularly in large and reasonably balanced datasets. In our study, the sample included 508 participants in the DAI group and 768 in the DAU group.

To validate the findings, we conducted the Wilcoxon Signed-Rank Test to compare fixation times between AV and non-AV settings within each group:

- For the DAI group, the difference in fixation times was statistically significant (p = 0.029).
- For the DAU group, the difference was highly significant (p = 0.0001).

These supplementary analyses confirm the robustness of the Two-Way ANOVA results and support the conclusion that fixation times differ significantly across scenario settings and groups. We have added this additional information and detailed explanations in Section 4.2.1, pages 25-26, lines 637-669, and lines 674-685.

4. For the result part, the paper analyzes the differences between the two methods in cognitive participation. However, for the conclusion of the study, for example, Figure 2 uses a visual attention heat map in a single scene to analyze the driver's attention difference under different conditions, but a single case is not representative. Should we consider counting all the experimental data and analyzing it by means of average attention, so as to enhance the persuasiveness of the results?

**Response**: We thank the reviewer for this observation. To clarify, Figure 2 is intended as an illustrative example, showing the fixation times on that specific scenario aggregated across all DAI and DAU participants who took part in the experiment. It is not presented as a representative analysis of all experimental data. However, Figure 3 (in-lab heatmap) shows aggregate data from DAI and DAU on all the scenarios, and the chosen scenario is only illustrative. Aggregating data from all the scenarios was possible only with our in-lab eye tracker software. We adjusted the manuscript accordingly to highlight this difference more clearly, in Section 4.2.1, page 27, lines 698-702, and in Section 4.2.2, page 28, lines 727-730 of the manuscript.

For a comprehensive analysis, Fig 1 (boxplots) represents the difference between DAI and DAU based on average fixation duration on the left side, and cumulative fixation duration on the right side. Additionally, we have calculated and reported the average attention across categories in Table 3 (online average fixation time per category) and Table 4 (in-lab average fixation time per category). These tables provide a robust summary of the differences in attention between AV and non-AV scenarios. Thus, Figure 2 serves as a visual illustration to complement these detailed analyses, highlighting the observed differences in attention patterns under specific conditions.

5. In the part of result analysis, the study found the differences of personal experience, knowledge in specific fields, social demography, culture, geography and online participants on the decision-making of the algorithm, but it did not classify and analyze what personal experience, demography and culture would have on the results, and the analysis of the results was weak, so it was suggested to add.

**Response**: We thank the reviewer for highlighting this important point. Our study has assumed, based on prior research, that imaginaries towards algorithmic decision-making may be shaped by diverse factors such as personal experiences, domain-specific knowledge, socio-demographics (Araujo et al. 2020), and cultural and geographic differences (Grzymek & Puntschuh 2019). While our findings suggest that these differences may play a role in shaping perceptions, we did not specifically classify and analyze the impact of individual factors such as personal experience, demography, or culture, as it was out of our scope in this study. However, this is our focus in the next analysis phases. We have added this additional clarification in the manuscript in Section 3, page 12, lines 347-349, and Section 5, page 36, lines 907-909.

To address the geographic variation further and due to the skewness of our online data (Fig 1), we have already analyzed whether the participant’s country impacted the results. The association between fixation times and participants’ countries was assessed using a chi-square test (page 27, lines 693-696), which revealed a statistically significant association (χ2 = 1284.06, df = 60, p < 0.001). This confirms that geographic location is indeed a factor influencing differences in fixation times. This analysis only scratches the surface of understanding that geography may shape perception. Due to the scope limitations of this article, we will explore this aspect in more depth in future studies, as our main aim was to test the suitability of online and in-lab eye-tracking methods in measuring perceptions towards automated decision-making.

We believe that this limitation highlights the need for future studies to delve deeper into the specific roles that these factors play in shaping imaginaries and perceptions. This was highlighted in Section 5.1, page 38, lines 963-970.
