# Supplementary material for: Sociotechnical imaginaries of autonomous vehicles: Comparing laboratory and online eye-tracking methods
Source: PLoS One. 2025 Nov 17;20(11):e0335672. doi: 10.1371/journal.pone.0335672 (PMC12622791; doi:10.1371/journal.pone.0335672)
Supplement: Attachment — Submitted filename: Response letter to Reviewers.docx [file pone.0335672.s005.docx]

Dear Dr. Quan Yuan,

We would like to express our sincere gratitude for the valuable feedback on our article *‘Sociotechnical Imaginaries of Autonomous Vehicles: Comparing Laboratory and Online Eye-Tracking Methods’.*

Please find attached our revised article and responses to the reviewers’ suggestions. We have marked the changes using ‘Track Changes,’ and our responses below are highlighted in blue.

Based on the reviewers’ recommendations, we have made minor changes to the reference list. The additions to the list of references were made with the aim of supporting the claims regarding the article’s methodological contribution and strengthening the discussions in relation to previous work.

Thank you for considering our article for publication. We look forward to your response.

Yours sincerely,

Mergime Ibrahimi

Anu Masso

Mauro Bellone

Reviewers’ comments:

Reviewer #2: The manuscript makes a valuable contribution by providing novel insights into how individuals perceive autonomous vehicles via complementary eye-tracking methodologies. With revisions addressing data transparency, methodological rigor, detailed statistical justification, and a more comprehensive discussion of limitations and implications, the paper would be suitable for publication.

1.The experimental design, integrating both in-lab and online studies, is well-conceived; however, the relatively small sample size in the laboratory setting (N=16) may limit the generalizability of the findings. This limitation should be more explicitly acknowledged and discussed.

**Response:** We thank the reviewer for highlighting this important issue. The relatively small sample size (N=16) for our in-lab experiment indeed presents limitations regarding the generalizability of the findings, especially if this were a standalone study. However, the role of the in-lab experiment in this study was to validate and complement our extensive online eye-tracking study. The large-scale study involved a significantly larger and more diverse participant sample. In the case of a small-scale study, the aim was not to obtain a representative sample, but rather to create a purposive or strategic sample (Suri, 2011) in order to ensure as diverse a range of perspectives as possible, allowing for the design of the present study and the testing of the developed method prior to conducting a larger-scale study. In addition, this comparison of different methods aims to contribute to existing methodological discussions, where increasing attention is being paid to online (semi-experimental) studies. This type of comparison will hopefully also provide a meaningful contribution to the design of future eye-tracking studies, both online and onsite.

To address this explicitly, we have clarified this purpose in the Method section (page 13-14, lines 274-291), Discussion sections (page 38, lines 801-804 and page 39 lines 837-840) and Limitation section (page 41-42, lines 887-897) in the manuscript. Furthermore, we add a sentence in the Method section (page 16, lines 340-342) to acknowledge that territorial representativeness remains a challenge in online crowd-sourced studies.

2.The discussion section should more thoroughly integrate the study’s findings with existing literature on sociotechnical imaginaries and automated decision-making. In particular, elaborating on the implications for AV design, public policy, and broader societal impacts would add significant value.

**Response:** We sincerely thank the reviewer for emphasizing the importance of contextualizing our findings within existing literature on sociotechnical imaginaries and automated decision-making. We have substantially revised the Discussion section (page 36, lines 753-757, and page 38-39, lines 811-824) to weave our empirical results into two key strands of scholarship: (a) sociotechnical imaginaries of automated decision-making and (b) methodological work using crowd-sourced online eye-tracking. We now open the Discussion by situating AV futures within the “dreamscapes of modernity” framework proposed by Jasanoff (2015), the promises to cure the “driver problem” by removing human error (Braun & Randell, 2020), and the “society-in-the-loop” challenge (Rahwan, 2018) and showing our contribution in demonstrating the potential of online crowd-sourced eye-tracking as a scalable method to capture diverse, global perceptions of AV technologies.

Then we framed our methodological contribution (page 39, lines 830-842). By adding references to other online eye-tracking literature and related crowd-sourced studies, we show how our study repurposes crowd-sourced eye-tracking from usability testing to the measurement of public imaginaries towards emerging technologies. Additionally, we have expanded our discussion (page 40-41, lines 848-878) by referencing specific policy frameworks, particularly highlighting the EU AI Act, to illustrate concrete implications for policymakers, AV designers and developers, and broader society.

3.Providing further justification for the statistical choices and exploring variance assumptions in greater depth would be beneficial.

**Response:** We appreciate the reviewer’s suggestion to clarify and justify our statistical approach more thoroughly. We have accordingly expanded our explanation of statistical methods (page 26-27), including a deeper discussion of variance assumptions related to our analytical choices. More specifically, we have now added an additional explanation to clarify that the ANOVA (lines 537-539) was chosen because it tests both main effects and their interaction and remains reliable with the large data sizes. We reported the effect-size (page 26, line 540 and page 27, line 543) – partial η² values are now given for the group effect (0.009) and the scenario effect (0.0005) to quantify the magnitude of each result. We added assumption checks (page 27, lines 552-556): 1) Shapiro–Wilk on a random 5 000-residual subset (W = 0.663, p <0 .001) is reported, and we briefly justify the 5 000-case subsample as the practical upper limit for the test; and 2) Levene’s test is now presented with its exact statistic (F(3, 17 860) = 24.72, p < .001). To demonstrate that our findings do not hinge on the violated assumptions, we added (page 27-28, lines 556-566) a Welch heteroscedastic two-way ANOVA (HC3 estimator) and a Gamma-log generalized linear model, both of which reproduced the group and scenario main effects and the null interaction. Furthermore, we now reported both versions of the Wilcoxon test (page 28, lines 568-573) to give a transparent view of the data’s robustness 1) fixation-event level (as in the original draft) maximizes statistical power by using every gaze event as a pair; this yields p = 0.029 for DAI and p = 0.0001 for DAU, and 2) participant-level means aggregate those events within individuals, eliminating the risk of pseudo-replication and providing a more conservative inference; here the DAI contrast becomes a non-significant trend (p = 0.105) whereas the DAU contrast remains significant (p = 0.044). Including both analyses demonstrates that the overall scenario effect is stable, while also signaling to readers how the choice of statistical unit affects the strength of the evidence, an important consideration for future replication and meta-analysis.

These insertions provide thorough statistical justification and variance exploration, while keeping the substantive conclusions unchanged.
